# Cytochrome P450 site of metabolism prediction from 2D topological fingerprints using GPU accelerated probabilistic classifiers

**DOI:** 10.1186/1758-2946-6-29

**Published:** 2014-05-27

**Authors:** Jonathan D Tyzack, Hamse Y Mussa, Mark J Williamson, Johannes Kirchmair, Robert C Glen

**Affiliations:** 1 Unilever Centre for Molecular Science Informatics, Department of Chemistry, University of Cambridge, Lensfield Road, CB2 1EW Cambridge, UK; 2 ETH Zurich, Department of Chemistry and Applied Biosciences, Institute of Pharmaceutical Sciences, HCI G 474.2, Vladimir-Prelog-Weg 1-5/10, 8093 Zurich, Switzerland

**Keywords:** Cytochrome P450, Metabolism, Probabilistic, Classification, GPU, CUDA, 2D

## Abstract

**Background:**

The prediction of sites and products of metabolism in xenobiotic compounds is key to the development of new chemical entities, where screening potential metabolites for toxicity or unwanted side-effects is of crucial importance. In this work 2D topological fingerprints are used to encode atomic sites and three probabilistic machine learning methods are applied: Parzen-Rosenblatt Window (PRW), Naive Bayesian (NB) and a novel approach called RASCAL (Random Attribute Subsampling Classification ALgorithm). These are implemented by randomly subsampling descriptor space to alleviate the problem often suffered by data mining methods of having to exactly match fingerprints, and in the case of PRW by measuring a distance between feature vectors rather than exact matching. The classifiers have been implemented in CUDA/C++ to exploit the parallel architecture of graphical processing units (GPUs) and is freely available in a public repository.

**Results:**

It is shown that for PRW a SoM (Site of Metabolism) is identified in the top two predictions for 85%, 91% and 88% of the CYP 3A4, 2D6 and 2C9 data sets respectively, with RASCAL giving similar performance of 83%, 91% and 88%, respectively. These results put PRW and RASCAL performance ahead of NB which gave a much lower classification performance of 51%, 73% and 74%, respectively.

**Conclusions:**

2D topological fingerprints calculated to a bond depth of 4-6 contain sufficient information to allow the identification of SoMs using classifiers based on relatively small data sets. Thus, the machine learning methods outlined in this paper are conceptually simpler and more efficient than other methods tested and the use of simple topological descriptors derived from 2D structure give results competitive with other approaches using more expensive quantum chemical descriptors. The descriptor space subsampling approach and ensemble methodology allow the methods to be applied to molecules more distant from the training data where data mining would be more likely to fail due to the lack of common fingerprints. The RASCAL algorithm is shown to give equivalent classification performance to PRW but at lower computational expense allowing it to be applied more efficiently in the ensemble scheme.

## Background

The prediction of sites and products of metabolism for xenobiotic and endogenous compounds is an important avenue of research, playing an influential role in the development and use of pharmaceuticals, cosmetics, nutritional supplements and agrochemicals. Toxicity of metabolites can play a major role in the withdrawal of new drugs or black-box warnings, contributing to the high attrition rates in the development of new chemical entities.

The cytochrome P450s (CYPs) are a family of heme-containing enzymes involved in the phase-I metabolism of over 90% of drugs on the market [1,2]. The CYP family of enzymes consists of 57 isoforms with the majority of biotransformations in mammals facilitated by the CYP 3A4 isoform, followed by 2D6 and 2C9.

The most common reactions catalysed by CYPs involve the insertion of a single oxygen into an organic molecule giving rise to C=C epoxidation, aromatic C oxidation, S oxidation and C-H hydroxylation reactions, the last example often leading to N-dealkylation or O-dealkylation if oxidation occurs on a suitable leaving group in an amine or ether moiety.

A host of computational approaches to predict SoMs have been developed as an alternative or aid to the resource and time consuming nature of experimental investigation. These approaches can be either ligand-based, where the structures and properties of known substrates or non-substrates are modelled to develop structure-activity relationships, or structure-based, where the structure of the metabolising CYP enzyme and its interactions with ligands are modelled. The reader is referred to the many comprehensive review papers [3-7] for an overview of the current computational tools to predict SoMs.

Many methods consider reactivity and accessibility factors since a SoM must be sufficiently reactive and also able to come into close proximity to the reactive heme centre. One such example is SMARTCyp [8], a Java-based SoM predictor that uses a database of activation energies for various pre-defined ligand fragments to assign reactivity estimates to matching moieties in a query ligand, with an accessibility descriptor used to tune the ranking.

Other methods take the accessibility consideration further and employ docking techniques to refine the predictions from reactivity approaches. Examples of these methods include IMPACTS [9], which combines docking with a fragment based reactivity approach, and a recently published approach [10] that makes use of a tethered docking methodology using GOLD [11] combined with a reactivity approach based on hydrogen bond order descriptors and a novel implementation of the average local ionisation energy.

In contrast to these approaches the methods described in this work do not require the explicit modelling of ligand binding or reactivity, but make use of machine learning techniques applied to an appropriate, representative data set. Various machine learning methods have been applied to the problem of SoM metabolism with some of the major contributions summarised below.

An example of a data-mining approach is MetaPrint2D [12], an online metabolism prediction tool trained on the Accelrys Metabolite Database [13] that makes SoM predictions based on occurrence counts of atomic fingerprints within the database where they appear as SoM versus non-SoM. If matching sites are not found in the database, Metaprint2D informs the user and makes no predictions rather than extrapolating beyond its domain of applicability.

Many methods employing machine learning techniques generate a wide-range of descriptors for each atomic site in the data set often including quantum chemical and electronic descriptors. An example is RegioSelectivity (RS)-predictor [14,15] which uses a Support Vector Machine (SVM) to predict SoM using 148 topological and 392 quantum chemical atomic descriptors where some of these descriptors are modified to include contributions from neighbouring atoms. A neural network approach called Xenosite [16] has also been applied to this descriptor set but combined with other molecular descriptors and fingerprint descriptors based on the Daylight [17] definition. A probability score that each atomic site is a SoM is obtained allowing the different sites in the molecule to be ranked with improved predictive performance over (RS)-predictor reported.

Another study [18] combines descriptors based on the electronic structure of the molecule with explicitly calculated activation energies [19,20], and also incorporates Solvent Accessible Surface Area (SASA) descriptors calculated using MOE [21]. Classification of the atomic sites in the data set into SoMs and non-SoMs was performed using random forest/ensemble decision trees, with the activation energy shown to be the most important in determining SoM.

A further method [22] with relevance to small endogenous molecules makes use of the Kyoto Encyclopedia of Genes and Genomes (KEGG) pathway database [23] where 4843 reactions were classified into 80 classes. SMARTS patterns were used to define chemical substructures (reaction centres and surrounding regions) with various descriptors used to encode these sites including electronic, energetic, topological, distance and steric. For each reaction centre a SVM binary classification model was trained and the score obtained for each potential SoM in a query molecule was used to rank the candidate reaction centres.

The computational expense of using quantum chemical descriptors is addressed by FAME [24], a metabolism prediction tool that applies random forest models to the Metabolite [13] database. It calculates atomic descriptors using the CDK [25] relating to charge and molecular topology and generates SoM predictions in a few seconds per molecule. FAME has other benefits since it is not just a predictor of CYP metabolism but reflects the broader enzyme reactions documented in the Metabolite database and can be filtered for Phase-I and Phase-II metabolism in human, rat and dog.

A recent publication [26] describes an approach to SoM prediction that applies the PASS algorithm to atom environment fingerprints encoded with 2D descriptors. This allows the method to be faster than those methods that must first generate 3D structures to calculate quantum chemical descriptors and gives results that are competitive with RS-Predictor and SMARTCyp.

This paper takes a similar approach using the 2D topological circular fingerprint [27,28] of atomic sites within a molecule as the sole descriptor making the method computationally less expensive than those that use 3D descriptors. Three probabilistic classifiers have been applied to the problem of xenobiotic SoM prediction: the Naive Bayesian (NB), the Parzen-Rosenblatt Window (PRW) and a novel method called RASCAL (Random Attribute Subsampling Classification Algorithm) that will be presented in the Methods section. In all classifiers the machine learning technique was applied on an ensemble basis by randomly subsampling descriptor space and treating each sub-classifier as one vote in an overall classification.

The kernelised nature of the PRW and RASCAL algorithms allied with the ensemble approach lends itself to a parallel implementation exploiting the massively parallel processing capability of Graphical Processing Units (GPUs). The computing industry is moving towards a parallel model as the limitations and capabilities of modern semiconductor manufacturing mean that ever increasing performance from a single processor is no longer possible [29]. In recent years, GPUs have become increasingly competitive with regard to programmability, speed and price, with the release of CUDA (Compute Unified Device Architecture) providing a standard C-like interface allowing scientists to exploit the parallel power of the GPU. The CUDA model operates by launching blocks of threads that are executed on stream multiprocessors (SMs) concurrently. Threads and blocks can be referred to by identification numbers allowing each to operate on a different portion of the data, where threads in a common block can communicate via a localised shared memory.

Recent developments in GPU accelerated classification tools include implementations for support vector machines [30-32], neural networks [33], k-nearest neighbours [34] and a parallel tool for the classification of remotely sensed imagery [35]. The techniques described in these studies could lead to similar efficiency gains when applied to cheminformatics classification problems and in the remainder of this paper GPU accelerated probabilistic classifiers are applied to the problem of SoM identification. The CUDA implementation released as part of this work could be applied to other binary or multi-class data sets, where in the case of RASCAL and NB the feature vectors would need to consist of integer or binary values. It is hoped that this implementation would be of interest to members of the cheminformatics community applying classification approaches to large data sets where performance is hindered by high computational demands.

In the Methods section the data sets and descriptors are presented, along with the three probabilistic classifiers (PRW, RASCAL and NB) and a discussion of the CUDA implementation. In the Results and discussion section the classification performance of the different methods is presented in terms of the Matthews Correlation Coefficient (MCC), area under the ROC curve and the percentage of the data sets where a SoM is identified in the top k positions. The effect of varying the size of the circular fingerprints used to describe atomic sites is investigated and a benchmarking analysis comparing the speed performance of the CUDA implementation on a Tesla C2075 GPU and a GeForce GT640 GPU to reference is presented. The important inferences from this work are presented in the Conclusions section.

To emphasize the benefits and novel aspects of this work it is important to point out that the SoM prediction models are built from 2D topological circular fingerprints without the requirement for complex quantum chemical and 3D descriptors. RASCAL combines the classification performance of the PRW with greater computational speed and hence could be applied to other much larger data sets. The data sets employed here are relatively small and show that SoMs can be identified within data sets of the order of 100’s of molecules being of potential interest to pharmaceutical companies with limited data on a specific series of molecules.

## Experimental

### Data sets

The machine learning approaches used in this study have been applied to the publicly available CYP 3A4, 2D6 and 2C9 data sets that originate from those initially released in the supporting information of the RS-Predictor paper [15] but further curated [9] with reference to the primary literature to identify and eliminate conflicting information. Open Babel v2.3.1 [36] was used to generate the protonation state of each ligand at pH 7.4 and the mol2 files generated are made available in the Additional files. The data sets have been sampled on a leave-one-out-cross-validation basis to generate results that are comparable with other reported methods [16].

**Figure 1:**
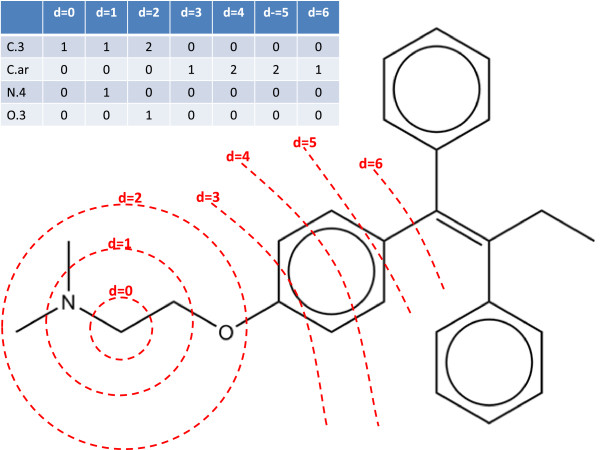
**Topological circular fingerprints.** Graphic to represent the construction of a circular fingerprint to a bond depth, d, of 6 using counts of SYBYL atom types at each level.

The feature vectors used to describe the atomic sites in each molecule are 2D topological circular fingerprints [27,28] based on the occurrence counts of SYBYL [37] atom types at different topological distances from the atom in question (see Figure 1 for a pictorial representation of the construction of these fingerprints). The size of the circular fingerprints can be varied by choosing different bond depths and for this study separate training sets were created for bond depths ranging from 0-8, thus allowing the impact of bond depth on classification performance to be assessed. A Java program was written to read in the data sets in mol2 [13] format and generate the circular fingerprints creating two classes: those circular fingerprints associated with a SoM and those not associated with a SoM. The circular fingerprints assigned to these two classes for each data set and bond depth were used as the inputs to the machine learning approaches described in this work. The data sets for each isoform for bond depths ranging from 4-6 are made available in the Additional files section in Additional files 1, 2, 3, 4, 5, 6, 7, 8 and 9. The mol2 files used to generate these circular fingerprints are also made available in the Additional files section in Additional files 10, 11 and 12.

### SoM prediction

**Figure 2:**
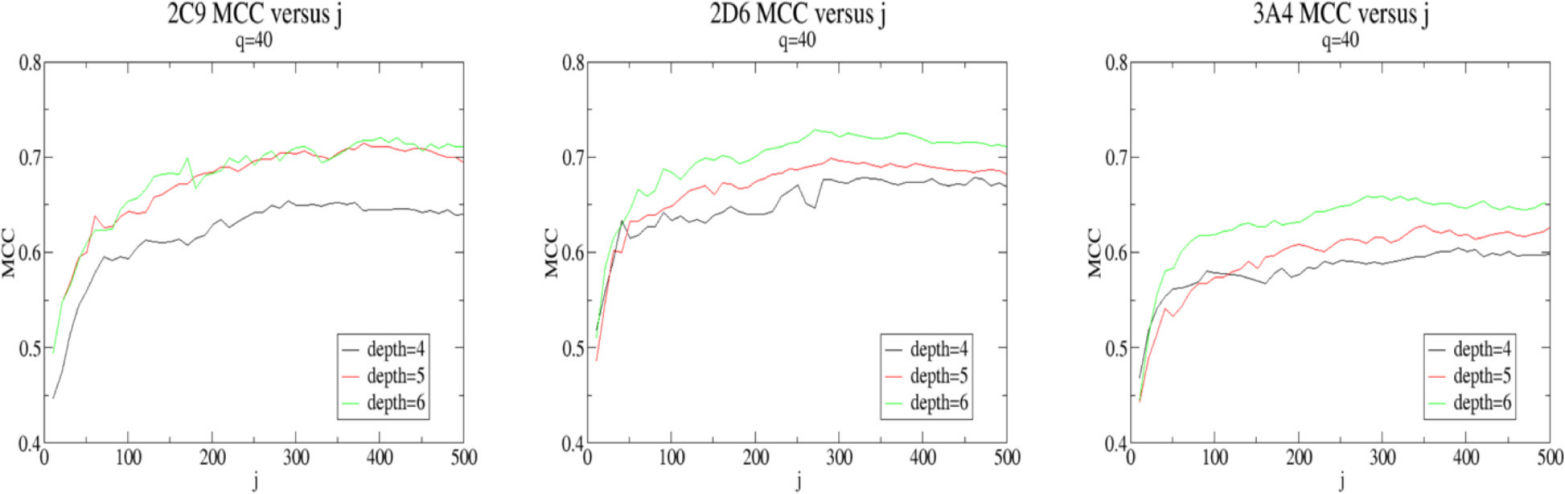
**Impact of number of subsamples, j.** Graphs showing classification performance for the three data sets in terms of MCC against number of subsamples *j* for models built using circular fingerprint bond depths ranging from 4-6. A value of *j*=201 was chosen to run the classification models.

The SoM classification performance of each approach described in the **Methods** section was assessed for each isoform data set using feature vectors calculated at each of the bond depths ranging from 0-8. These methods have been implemented on an ensemble subsampling methodology, hence as described previously the length of the subsample, *q*, needs to be optimised. Therefore, for each classification model *q* was varied from an initial value of 5 to the full number of features *L* in increments of 5, with the Matthews Correlation Coefficient (MCC) [38] and the area under ROC curves used to compare classification performance. Selection of the *j* parameter is a balance between sufficiently sampling feature space versus computational load, with a value of 201 found to be suitable in this case. The graphs shown in Figure 2 show classification performance against *j* for models with *q*=40 and bond depth ranging from 4-6 and show that by *j*=201 the classification performance has largely reached a plateau.

In this way the probability that an atomic site is a SoM can be calculated allowing all sites in a molecule to be ranked. The percentage of the data set where a SoM is identified in the top two (top-2%) and top three (top-3%) predictions was calculated to allow comparisons to other SoM identification methods. When calculating the top-2% and top-3% values steps were taken to identify atoms in equivalent sites to ensure that such sites are only included once in predictions. This was achieved by generating full circular fingerprints for each atomic site in the same way as described previously but this time spanning the entire ligand, with equivalent atomic sites defined as those with identical circular fingerprints.

### CUDA implementation

The three probabilistic classifiers have been implemented using the Nvidia Nsight [39] integrated development environment for CUDA/C++ released as part of CUDA Tools 5.5. A benchmarking analysis has been carried out comparing the performance speed of an Nvidia C2075 Tesla GPU and an Nvidia GT640 GeForce GPU against a reference CPU implementation running on a single Intel Xeon E5506 at 2.13GHz.

The source code is available in a public repository (https://bitbucket.org/jdt42/probclassifier_cuda) and could be applied to other classification problems where the input files are formatted in the appropriate manner for this tool. The code exploits the parallel nature of GPU’s by aligning the dimensions of the classification problem, such as number of feature vectors *N* and number of subclassifiers *j*, with the parallel architecture of CUDA where computational work is kernelised and spread over blocks of threads. The code makes use of atomic operations and so requires a CUDA enabled GPU with a compute rating of ≥ 1.1.

## Methods

### Probabilistic classifiers

Cheminformatics classification approaches that compare structural similarity to known molecules tend to be more successful if they allow for some degree of uncertainty (fuzziness) [40]. This means one treats the attributes representing the data items under study, and the class they are associated with as stochastic variables. In this scenario the classification problem can be viewed as a hypothesis testing task; this requires estimates of the probability densities of the attributes for each class. Estimates of these probability densities, coupled with an appropriate decision rule, constitute what is commonly referred to as statistical (or probabilistic) classifiers [40,41].

In the probabilistic pattern recognition framework, it is assumed that there is an unknown probability distribution *p* that underlies any relationship in the available data, *D*, where the data points belonging to *D* are drawn from a product space *X*×*Y* where *X* represents input patterns/objects and *Y* represents the class space. In this setting, the purpose of a learning algorithm is to discover the probability distribution *p* that captures the “functional” relationships that may exist between *X* and *Y*. In a typical pattern classification scenario the available data set *D* is finite and is defined as $D={\left\{({\text{x}}_{i},y_{i})\right\}}_{i=1}^{N}$, where **x**_*i*_∈*X*; *y*_*i*_∈*Y* and *N* refers to the size of the given sample data.

The process of finding an appropriate probabilistic classification model *p*(**x**_*i*_,*y*_*i*_) involves relating **x**_*i*_ probabilistically to its associated *y*_*i*_, where *p*(**x**_*i*_,*y*_*i*_) refers to the probability of (**x**_*i*_, *y*_*i*_) occurring.

In practice, we are often interested in the posterior probability that a given pattern **x**_*i*_ is associated with *y*_*i*_. The essence of our task is to generate an algorithm that is capable of inferring probabilistic classification rules from the given training set with the ability to generalise to new patterns. The input pattern **x**_*i*_ is an *L*–dimensional mathematical vector that inhabits an abstract *L*–dimensional space. The pattern vector **x**_*i*_ consists of *L* elements **x**_*i*_ = (*x*_*i*1_,*x*_*i*2_,…,*x*_*i* *L*_) that represent the information we have about *L* different but relevant properties of the *i*^*t* *h*^ pattern. The class label *y*_*i*_ often denotes a set of predefined classes {*ω*_0_,*ω*_1_,…,*ω*_*M*-1_}.

In this work we are concerned with classification problems where the elements *x*_*i* *l*_ can assume integer values being the counts of SYBYL atom types at specific bond depths from the atom described by **x**_*i*_. For the sake of clarity the index *i* in both **x**_*i*_ and *y*_*i*_ will be dropped in the rest of the paper; unless otherwise stated.

Bayes’ theorem allows us to compute *p*(**x**,*ω*_*α*_) (*i.e.*, *p*(**x**,*y*)) from a priori and conditional probabilities. This means that the class posterior probability *p*(*ω*_*α*_|**x**) of a given pattern **x** being associated with *ω*_*α*_[41] can expressed in the form [41-44]:

(1) $$p(\omega_{\alpha }|\text{x})=\frac{p(\text{x},\omega_{\alpha })}{p(\text{x})}=\frac{p(\omega_{\alpha })p(\text{x}|\omega_{\alpha })}{p(\text{x})}$$

where *p*(*ω*_*α*_) is the a priori probability of class *ω*_*α*_ and can be estimated from the class proportions in the training data set; *p*(**x**|*ω*_*α*_) is the class conditional probability of **x** belonging to class *ω*_*α*_; and *p*(**x**) is given by:

(2) $$p(\text{x})=\overset{M-1}{\underset{\alpha =0}{\sum }}p(\omega_{\alpha })p(\text{x}|\omega_{\alpha })$$

To make a probabilistic classification on a new pattern **x**, the probability of **x** belonging to each class *ω*_*α*_ needs to be computed, with **x** assigned to the class with the highest probability value:

$$\omega_{\alpha }(\text{x})=\underset{\alpha^{\prime }\in (0,1,\ldots ,\alpha ,\ldots ,M-1)}{\arg\;\max}P(\omega_{\alpha^{\prime }}|\text{x})$$

The prediction efficiency of the generated classifier can be validated by comparing classification predictions made by the model with actual known classes. Once the model is deemed reliable, it can then be used to make predictions about the behaviour of real world patterns coming from the same domain, *X*, as the given training set.

Clearly the estimation of the probability function *p*(**x**|*ω*_*α*_) is the most important step in the estimation of *p*(*ω*_*α*_|**x**), *vide supra*. Thus, over the years a number of approaches to obtain estimates of *p*(**x**|*ω*_*α*_) have been developed [44-46].

When the data are sparse in the descriptor/input space, it can become difficult to generate a good estimate of *p*(**x**|*ω*_*α*_). This may result in a probabilistic classifier (a single classifier) with poor generalisation performance. This problem is also encountered when one tries to construct non–probabilistic classifiers on a data set sparse in the descriptor space.

There are different approaches that can be used to improve the generalisation performance of single classifiers. Feature selection schemes [41]; regularisation methods [42,46]; and the so–called ensemble learning technique (which is currently popular [42,47,48]) are good examples of these approaches. In the ensemble technique, the scheme employed in this work, the classification of a new pattern **x** is typically made through majority voting of the classification predictions of *j*(>1) single (or base) classifiers. Empirical and theoretical results indicate that an ensemble of base classifiers gives improvements in generalisation over the individual classifiers [49], providing the base classifiers are not correlated with one another. It was suggested that an effective method of achieving uncorrelated individual classifiers in an ensemble is by training the single classifiers using randomly selected *q* distinct attributes/features of the available *L* features where *q*≤*L*[49] and the *q*–dimensional descriptor vector is denoted by **x**^*q*^.

It is important to note that, in this ensemble approach, the number of training data points remains the same, *i.e.*, *N*. Thus, the relative (with respect to *q*, the descriptor subspace dimension) training sample size increases [50], which in turn can improve the approximation of the class–conditioned probability from the training set.

Three probabilistic classifiers based on the ideas briefly described in the preceding paragraphs have been used to create SoM prediction tools, namely the Naive Bayesian (NB) [41,42], Parzen–Rosenblatt Window (PRW) [51] and an internally developed methodology called RASCAL. The latter two approaches are kernel based, *vide infra*.

In the following sections we describe how *p*(*ω*_*α*_|**x**^*q*^) is estimated in each of the classification methods. The atomic site and its class (SoM or nonSoM) are represented by **x**^*q*^, and *ω*_*α*_, respectively. Note that in this work *M*=2, *i.e.* we are only dealing with 2 classes *ω*_0_ and *ω*_1_, where *ω*_0_ refers to SoM and *ω*_1_ denotes nonSoM. Finally it is assumed that all a priori class distributions are equal, i.e., *p*(*ω*_0_)=*p*(*ω*_1_).

### Naive Bayesian (NB)

In the Naive Bayesian case, the class conditional *p*(**x**^*q*^|*ω*_*α*_) can be estimated as

(3) $$p({\text{x}}^{q}|\omega_{\alpha })=\overset{q}{\underset{l=1}{\prod }}p(x_{l}^{q}|\omega_{\alpha })$$

where

(4) $$p(x_{l}^{q}|\omega_{\alpha })=\frac{C_{l}+1}{N_{\alpha }+2}$$

with *C*_*l*_ being the number of times descriptor $x_{l}^{q}$ assumes the same value in class *ω*_*α*_ and *N*_*α*_ is the number of training items belonging to class *ω*_*α*_.

Since *p*(*ω*_0_)=*p*(*ω*_1_), the posterior probability prediction for membership of a particular class is computed as

(5) $$p(\omega_{\alpha }|{\text{x}}^{q})=\frac{p({\text{x}}^{q}|\omega_{\alpha })}{\sum_{\alpha }p({\text{x}}^{q}|\omega_{\alpha })}$$

The data item **x**^*q*^ is predicted to be in the class with the highest posterior probability *p*(*ω*_*α*_|**x**^*q*^), and in the case where equal posterior probabilities are calculated for two classes they are ranked arbitrarily. The final class membership probabilities are calculated as the ratio of the number of votes for that class divided by the total number of subclassifiers *j*.

### Kernel based probabilistic classifiers

In kernel based methods, the average similarity, $S_{\alpha }^{i}$, of a test atom ${\text{x}}_{i}^{q}$ with the set of atoms ${\text{x}}_{k}^{q}$ in the training set for class *ω*_*α*_ is calculated by comparing ${\text{x}}_{i}^{q}$ with all examples in the training set for *ω*_*α*_ as

(6) $$S_{\alpha }^{i}=\frac{1}{N_{\alpha }}\underset{{\text{x}}_{k}^{q}\in \omega_{\alpha }}{\sum }K({\text{x}}_{i}^{q},{\text{x}}_{k}^{q})$$

where *N*_*α*_ represents the number of training data items belonging to class *ω*_*α*_ and the kernel function $K\left({\text{x}}_{i}^{q},{\text{x}}_{k}^{q}\right)$ measures the similarity between ${\text{x}}_{i}^{q}$ and ${\text{x}}_{k}^{q}$. In this case the class conditional probability $p\left({\text{x}}_{i}^{q}|\omega_{\alpha }\right)$ is equal to $S_{\alpha }^{i}$, *i.e.*

(7) $$p({\text{x}}_{i}^{q}|\omega_{\alpha })=\frac{1}{N_{\alpha }}\underset{{\text{x}}_{k}^{q}\in \omega_{\alpha }}{\sum }K\left({\text{x}}_{i}^{q},{\text{x}}_{k}^{q}\right)$$

Two different kernel functions have been implemented: the PRW and a Dirac kernel [52] used in the implementation of RASCAL, both described in the next subsections.

### Randomised attribute subsampling classification algorithm (RASCAL)

In this study we present a probabilistic classifier called RASCAL that uses a Dirac kernel function as shown below.

(8) $$K({\text{x}}_{i}^{q},{\text{x}}_{k}^{q})=\left\{\begin{array}{c} 1\;\;\;{\text{x}}_{i}^{q}={\text{x}}_{k}^{q}\\ 0\;\;\;{\text{x}}_{i}^{q}\neq {\text{x}}_{k}^{q} \end{array}\right)$$

In the case where the descriptors are defined over the binary domain it has been shown that this kernel is equivalent to a full expansion in Radmacher Walsh polynomials [53]. However, in this application the circular fingerprints used to describe the atomic sites are integer valued although the closed form of the kernel shown above can be applied to all discrete valued feature vectors.

### Parzen-Rosenblatt window (PRW)

In this study, a Gaussian kernel has been used for PRW defined as

(9) $$K({\text{x}}_{i}^{q},{\text{x}}_{k}^{q})=\frac{1}{{\left(h\sqrt{2\pi }\right)}^{q}}\exp\left(-\frac{{\left({\text{x}}_{i}^{q}-{\text{x}}_{k}^{q}\right)}^{T}({\text{x}}_{i}^{q}-{\text{x}}_{k}^{q})}{2h^{2}}\right)$$

where ${\left({\text{x}}_{i}^{q}-{\text{x}}_{k}^{q}\right)}^{T}({\text{x}}_{i}^{q}-{\text{x}}_{k}^{q})$ is a measure of the distance between ${\text{x}}_{i}^{q}$ and ${\text{x}}_{k}^{q}$, *q* is the number of features and *h* is a smoothing parameter, where a value of *h* = 0.1 was found to give good performance.

## Results and discussion

**Figure 3:**
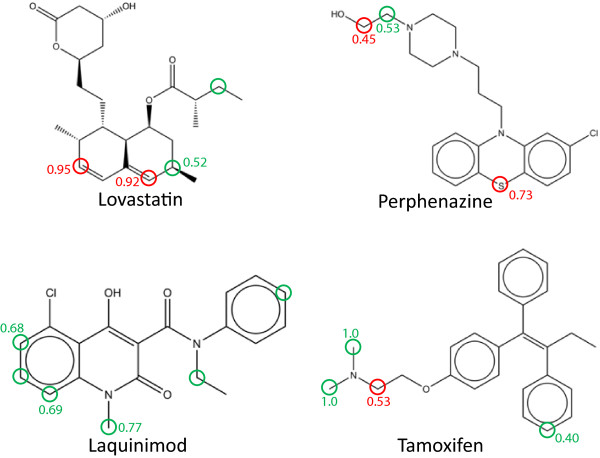
**Example SoM predictions.** Graphic to summarise the classification results for four molecules chosen at random from the CYP 3A4 data set using the scenario with a bond depth of 4, *q*=45, *j*=201 and the RASCAL classifier. SoMs have been labelled with a green circle and the scores for the top three predictions are labelled against the atom: green text is used where a SoM is identified, red text is used with a corresponding red ring where a non-SoM is identified.

Figure 3 summarises the classification results for four molecules chosen at random from the CYP 3A4 data set using the scenario with a bond depth of 4, *q*=45, *j*=201 and the RASCAL classifier. SoMs have been labelled with a green circle and the scores for the top three predictions are labelled against the atom: green text is used where a SoM is identified, red text is used with a corresponding red ring where a non-SoM is identified. A SoM is identified in the top 3 predictions for all molecules, and the top 2 predictions for all except Lovastatin, reflecting the strong classification performance observed across the entire data set.

### Circular fingerprint depth analysis

**Figure 4:**
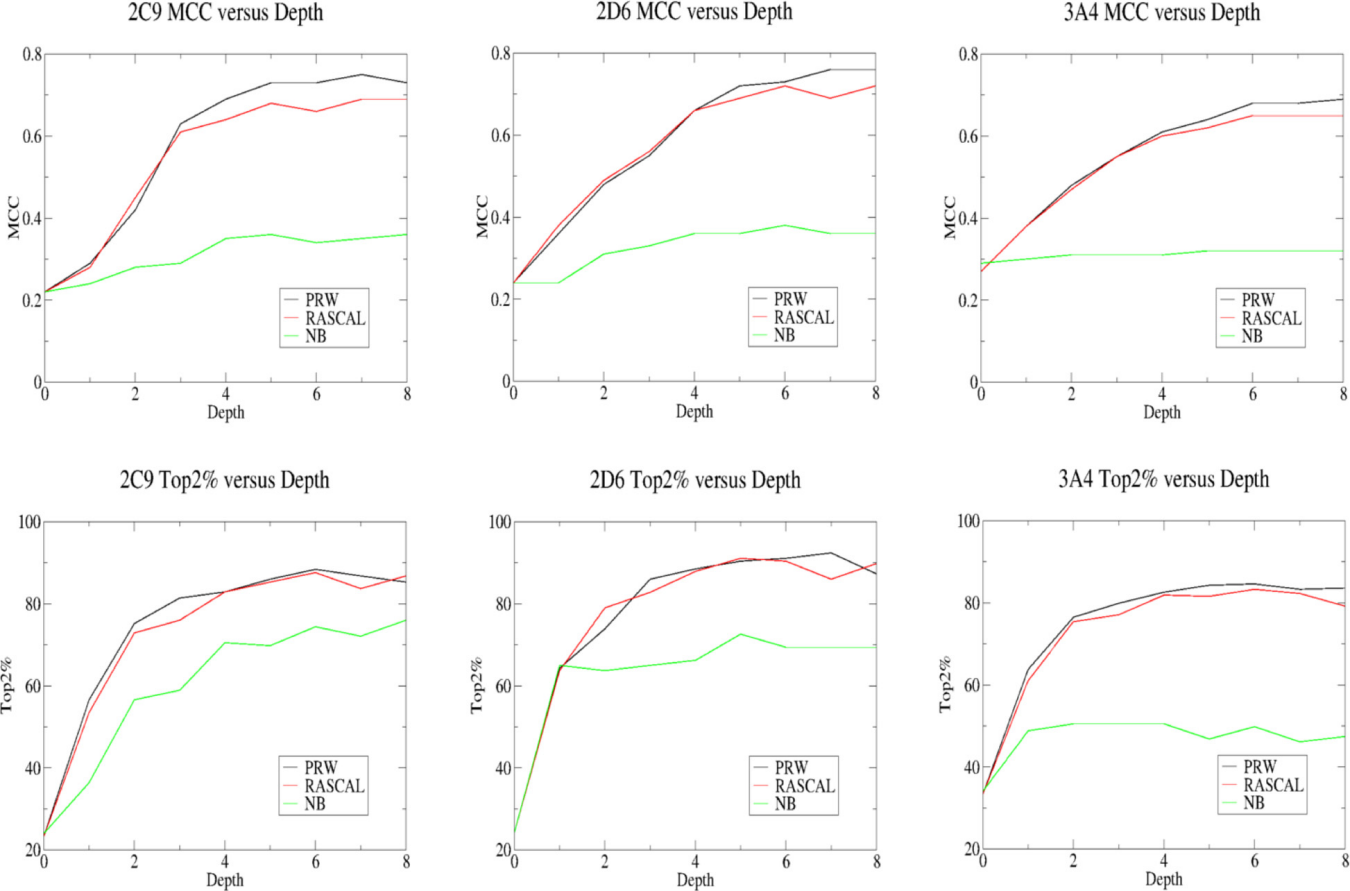
**Prediction performance in terms of MCC and top-2%.** Graphs showing classification performance in terms of MCC and top-2% (the percentage of the data sets where a SoM is identified in the top 2 atom positions) for the three data sets against the circular fingerprint bond depth used to build the models. The number of iterations *j*=201.

The graphs in Figure 4 show classification performance for the three data sets against the bond depth used to generate topological circular fingerprints, with the first row of graphs showing the Matthews Correlation Coefficient (MCC) and the second row of graphs showing the top-2%. Classification performance on an MCC basis is shown in Table 1 and generally improves with circular fingerprint bond depth since a more detailed description of each atomic site is being used, although the rate of improvement tends to slow with bond depth showing that the more local descriptors are most important. Classification performance on a top-2% basis shows similar trends although a plateau in classification performance tends to be reached earlier at a bond depth of 4-5. Table 2 shows the classification performance for circular fingerprint bond depths of 4-6 and it can be seen that they encode sufficient information for PRW and RASCAL top-2% performance of over 82% for all 3 isoforms, results that are competitive with other published methods [14,15], see Table 3.

**Table 1:** Site of metabolism prediction results, MCC and AUC

**Data**	**Bond**		**MCC**				**AUC**		
**set**	**depth**		**PRW**	**RASCAL**	**NB**		**PRW**	**RASCAL**	**NB**
	4		0.69	0.64	0.35		0.94	0.95	0.85
2C9	5		0.73	0.68	0.36		0.96	0.95	0.87
	6		0.73	0.66	0.34		0.97	0.96	0.87
	4		0.66	0.66	0.36		0.95	0.96	0.85
2D6	5		0.72	0.69	0.36		0.97	0.96	0.84
	6		0.73	0.72	0.38		0.97	0.97	0.84
	4		0.61	0.60	0.31		0.94	0.94	0.80
3A4	5		0.64	0.62	0.32		0.95	0.94	0.80
	6		0.68	0.65	0.32		0.96	0.94	0.81

Table shows the prediction results in terms of the Matthews Correlation Coefficient (MCC) and area under the ROC curve.

**Table 2:** Site of metabolism prediction results, top-k%

**Data**	**Bond**		**Top-3%**				**Top-2%**				**Top-1%**		
**set**	**depth**		**PRW**	**RASCAL**	**NB**		**PRW**	**RASCAL**	**NB**		**PRW**	**RASCAL**	**NB**
	4		92	88	81		83	83	71		71	71	39
2C9	5		87	88	81		86	85	70		76	75	43
	6		92	90	85		88	88	74		77	76	50
	4		92	94	79		89	88	66		66	77	53
2D6	5		93	92	83		90	91	73		73	78	45
	6		95	93	78		91	90	69		77	78	46
	4		89	89	64		83	82	51		67	69	26
3A4	5		89	85	61		84	82	47		69	69	28
	6		89	87	64		85	83	50		72	70	27

Table shows the prediction results in terms of the % of the data sets where a SoM is identified in the top-k predictions (top-k%).

**Table 3:** Comparison to other methods

**Data**		**Top-2%**						
**set**		**PRW**	**RASCAL**	**Xenosite**	**RSPredictor**	**SMARTCyp**	**Reactivity &**	**Random**
		**(depth=6)**		[16]	[14]**,**[15]	[8]	docking [10]	
2C9		88	88	87	85	86	78	18
2D6		91	90	89	86	84	80	22
3A4		85	83	88	82	80	75	21

Table shows site of metabolism prediction results in terms of the top-2% compared to other methods.

### Analysis of subsample length on classification for bond depths of 4-6

**Figure 5:**
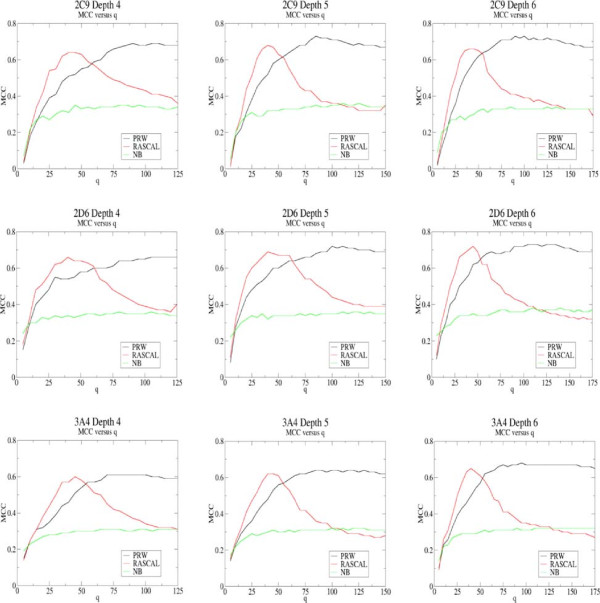
**Impact of subsample length, q.** Graphs showing classification performance for the three data sets in terms of MCC against subsample length *q* for models built using circular fingerprint bond depths ranging from 4-6. The number of iterations *j*=201. RASCAL requires careful parametrisation of *q* due to its sensitivity to this parameter.

**Figure 6:**
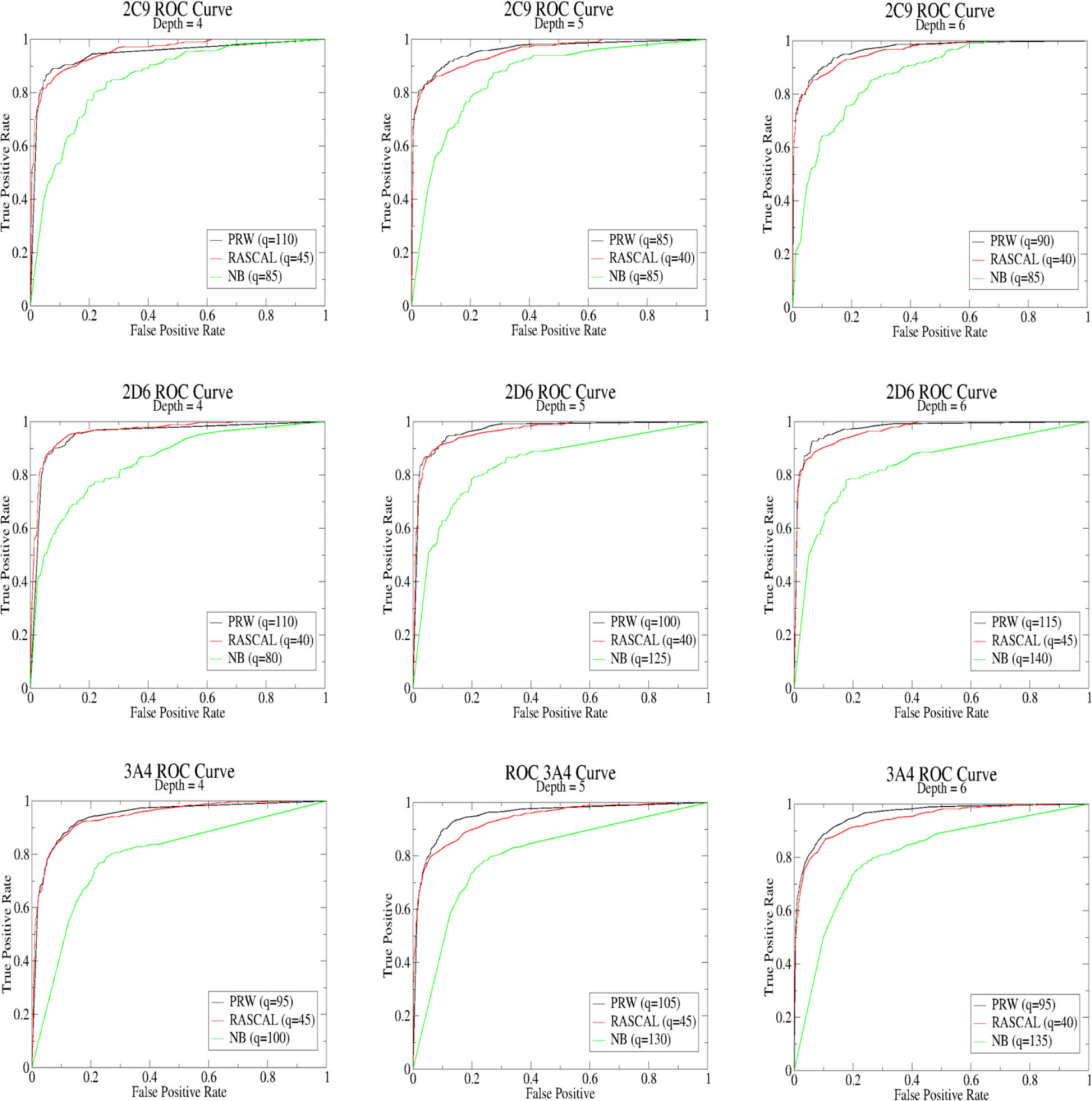
**ROC curves.** Graphs showing ROC curves for the three data sets using the best classification models built using circular fingerprint bond depths ranging from 4-6.

As described previously classification performance is dependent on the subsample length *q* and the number of subclassifiers *j*. The graphs in Figure 5 show the classification performance in terms of MCC against *q* for each of the models based on circular fingerprint bond depths ranging from 4-6. It can be seen that PRW and NB are less sensitive to *q* than RASCAL, which is to be expected since RASCAL is in effect a subsample matching algorithm and if the subsample becomes too long then no matching instances will be found in the training set for either class. Therefore when classifying a data set using RASCAL it is necessary to parametrise carefully to find a suitable value for *q*. For PRW and NB classification performance tends to plateau at high *q* when applied to these data sets and using a PRW smoothing parameter of *h*=0.1. For these approaches the extra computational cost of an ensemble approach is not justified in terms of improved performance and a standard implementation running over all *L* descriptors would be more suitable. It can be seen that PRW and RASCAL give similar classification performance once the parameter *q* has been optimised, with both systematically outperforming the NB. This is reinforced by the ROC curves shown in Figure 6 where PRW and RASCAL performance tracks above that for NB.

### Significance of molecular similarity

Machine learning becomes more challenging when test data is more dissimilar to training data. To investigate the ability of these methods to be applied to molecules more dissimilar from the training data a test set (TS1) representing 20% of each isoform data set was selected at random with the remaining 80% forming the training set. A second test set (TS2) was then defined as a 50% subset of TS1 representing the molecules most dissimilar to the training set, with Open Babel [36] used to measure molecular similarity using the default fingerprint. The SoM prediction performance is shown in Table 4 and shows that a worsening in performance in TS2 is more pronounced in the CYP 3A4 data set for the RASCAL classifier and on a top-2% basis in the CYP 2D6 data set for the PRW classifier indicating that care must be taken when extrapolating into chemical space more dissimilar to the training data although reasonable predictive performance is maintained.

**Table 4:** Impact of molecular similarity on prediction performance

**Data**	**Classifier**	**Bond**		**Top-3%**			**Top-2%**	
**set**		**depth**		**TS1**	**TS2**		**TS1**	**TS2**
2C9	PRW	5		85	85		82	85
		6		83	85		81	85
	RASCAL	5		85	85		78	85
		6		81	85		77	85
2D6	PRW	5		90	92		85	75
		6		91	94		86	79
	RASCAL	5		90	90		84	75
		6		87	88		84	83
3A4	PRW	5		83	82		80	78
		6		84	81		80	79
	RASCAL	5		80	76		76	67
		6		79	75		72	63

Table shows the classification performance in terms of top-2% and top-3% for test set 1 (TS1: 20% of each isoform data set selected at random) and test set 2 (TS2: the 50% of molecules in TS1 most dissimilar to the training data).

### Benchmarking analysis

A comparison of the running times of the PRW, RASCAL and NB algorithms on the three different platforms is given in Table 5 and the benefits from running in parallel are apparent. The results discussed previously have shown that RASCAL gives classification performance equivalent to PRW and so to become relevant and competitive as a classifier it is necessary to demonstrate that RASCAL is a faster more efficient algorithm. The relevant factors regarding the computational expense of the RASCAL and PRW algorithms are discussed next.

**Table 5:** Benchmarking analysis

**Data**	**Method**	**q**		**Runtime seconds**				**x-fold to Ref**	
**set**				**Ref**	**GT640**	**C2075**		**GT640**	**C2075**
	RASCAL	40		42	11	2.2		4	19
	NB	40		8.8	1.4	0.3		6	29
2C9	PRW	40		436	114	32		4	14
	NB	175		8.2	1.3	0.3		6	27
	PRW	175		7.0	31	3.6		0.2	2
	RASCAL	40		57	15	3.0		4	19
	NB	40		12	1.9	0.4		6	30
2D6	PRW	40		727	157	44		5	17
	NB	175		11	1.8	0.4		6	28
	PRW	175		9.7	43	5.0		0.2	2
	RASCAL	40		298	66	13		5	23
	NB	40		55	8.3	1.8		7	31
3A4	PRW	40		3,654	714	201		5	18
	NB	175		53	8.1	1.8		7	29
	PRW	175		45	197	23		0.2	2

Table shows the time taken in seconds and relative performance o the reference (Ref) to run all three classifiers over the three data sets using a scenario with bond depth of 6 (175 features) and *j* = 201 and *q* = 40. The reference is a single Intel Xeon E5506 at 2.13GHz. The number of vectors in data sets CYP 2C9, 2D6 and 3A4 are 2905, 3399 and 7294, respectively.

The reminder of this section makes use of the following variables to describe the computational expense of the various algorithms: *N* refers to the size of the training set for a particular class; *j* refers to the number of subsamples; *q* refers to the subsample size; and *L* refers to the full length of the feature vector. Using these definitions the PRW algorithm computational expense when subsampling descriptor space, PRW_**sub**_, can be estimated as

$$O({\text{PRW}}_{\text{sub}})\approx N\times j\times (3\times q+2)$$

whereas the computational expense of RASCAL can be estimated as

O(RASCAL)≈N×j×q

It should be noted that the RASCAL estimate is an upper bound. Only in the case where the training and test subsamples are identical are all *q* features compared otherwise the feature by feature comparison is halted as soon a difference is noted. Therefore the 15-fold faster running time of RASCAL over PRW can be justified by the lower computational expense, the early termination of the RASCAL kernel where relevant and the simpler nature of the RASCAL kernel compared to the PRW kernel.

However, as demonstrated in the Results and discussion section there is no benefit to performing subsampling with PRW, hence a more valid evaluation of algorithm running times is by comparison against PRW running over all *L* features without subsampling, PRW_**all**_, where the computational expense can be estimated as

$$O({\text{PRW}}_{\text{all}})\approx N\times (3\times L+2)$$

Therefore RASCAL is more likely to run faster than PRW when *q*≪*L*. In the data set with bond depth of 6, where *L*=175, applying RASCAL with *q*=40 and *j*=201 gives a speed increase of 1.5-2.0 compared to PRW running over all features. However, the case for using RASCAL is likely to become much more compelling when using data sets consisting of longer feature vectors where it is more likely that RASCAL models can be used where *q*≪*L*.

NB runs faster than RASCAL since it is possible to precompute the counts of common features between test data items and each training class so that the training data items only need to be parsed once, albeit at the price of poorer classification performance.

## Conclusions

The probabilistic classifiers outlined in this paper based on 2D topological fingerprints give SoM predictive performance that is competitive with other machine learning methods that employ complex 3D descriptors, enabling conceptually simple and efficient classification models to be built. Data mining approaches often suffer when fingerprints from a test molecule are not contained in the training data, but descriptor space subsampling and the use of classifiers that measure a distance between vectors instead of exact matching help to alleviate this problem. This enables the creation of classification models that can be based on relatively small data sets but still applicable to molecules more distant from the training data where data mining would be more likely to fail due to the lack of common fingerprints. Hence the methods could be of interest to pharmaceutical companies studying a series of molecules with only of the order of 100’s of data points available.

RASCAL and PRW were found to give similar predictive performance with PRW identifying a SoM in the top 2 predictions for 85%, 91% and 88% for the CYP 3A4, 2D6 and 2C9 data sets respectively, whereas for RASCAL the figures are 83%, 91% and 88%, respectively. This performance is competitive with other published machine learning methods [14]**,**[15] but is achieved using 2D descriptors. This suggests that there are common patterns in the local structure of SoMs in the data sets that are captured by the atomic circular fingerprints and can be identified by machine learning methods.

RASCAL gives similar classification performance to PRW but at lower computational expense making it suitable for use on large data sets and particularly for the ensemble schemes outlined in this paper. The speed boost from using RASCAL is likely to become more apparent for classification problems where *q*≪*L* (where *q* is the subsample length and *L* is the full length of the feature vector) and in these situations the benefits from using RASCAL are likely to become more compelling.

The suitability of using CUDA/C++ to exploit the parallel capabilities of GPU hardware for classification problems has been demonstrated and it is hoped that the source code will be of use to other researchers in the field. Further enhancements to the implementation could include maximising the use of faster memory (shared, texture and constant), implementing task parallelism using streams and implementing code to run on multiple GPUs, all of which should further improve the speed-up in performance compared to the reference.

In summary it has been shown that probabilistic classifiers implemented using randomly selected subclassifiers on an ensemble basis using 2D topological circular fingerprints as descriptors can give strong SoM predictive performance. However, as with all machine learning methods, the models are likely to be most relevant within their domain of applicability and are likely to perform less well against novel molecules that are very different from those in the training set.

## Competing interests

The authors declare that they have no competing interests.

## Authors’ contributions

JDT wrote the programs in C++ and CUDA, produced the data sets of circular fingerprints to represent the atomic sites and generated the results; HYM provided the theoretical ideas behind the probabilistic classifiers; MJW provided guidance with the programming and implementation and assistance with optimising the code; JK and RCG provided overall scientific guidance in the development of this work particularly around the use of atomic fingerprint descriptors. All authors read and approved the final manuscript.

## Supplementary Material

Additional file 1 Topological circular fingerprints for atomic sites in the 3A4 data to a bond depth of 4. Click here for file

Additional file 2 Topological circular fingerprints for atomic sites in the 3A4 data to a bond depth of 5. Click here for file

Additional file 3 Topological circular fingerprints for atomic sites in the 3A4 data to a bond depth of 6. Click here for file

Additional file 4 Topological circular fingerprints for atomic sites in the 2D6 data to a bond depth of 4. Click here for file

Additional file 5 Topological circular fingerprints for atomic sites in the 2D6 data to a bond depth of 5. Click here for file

Additional file 6 Topological circular fingerprints for atomic sites in the 2D6 data to a bond depth of 6. Click here for file

Additional file 7 Topological circular fingerprints for atomic sites in the 2C9 data to a bond depth of 4. Click here for file

Additional file 8 Topological circular fingerprints for atomic sites in the 2C9 data to a bond depth of 5. Click here for file

Additional file 9 Topological circular fingerprints for atomic sites in the 2C9 data to a bond depth of 6. Click here for file

Additional file 10 Ligands in the 3A4 data set in mol2 format. Click here for file

Additional file 11 Ligands in the 2D6 data set in mol2 format. Click here for file

Additional file 12 Ligands in the 2C9 data set in mol2 format. Click here for file
